# Sociodemographic correlates of depressive symptoms: a cross-sectional analytic study among healthy urban Ghanaian women

**DOI:** 10.1186/s12889-018-6322-8

**Published:** 2019-01-10

**Authors:** Harriet Affran Bonful, Adote Anum

**Affiliations:** 1 Department of Epidemiology and Disease Control, School of Public Health, College of Health Sciences, University of Ghana, Legon, Ghana; 2 Department of Psychology, University of Ghana, P O Box LG84, Legon, Accra, Ghana

**Keywords:** Depressive symptoms, Sociodemographic, Ghana, Accra, Women

## Abstract

**Background:**

Studies on healthy individuals that show minor signs of distress and depression—but that are not significant enough to be debilitating or to report to the hospital for treatment—are rare. Our primary objective was to measure the prevalence of depressive symptoms and sociodemographic correlates among healthy women 18 years and above in urban Accra, Ghana.

**Method:**

We used secondary data from the Women’s Health Study of Accra, Wave 1 (WHSA-1), a large scale, analytic, cross-sectional study conducted in Accra, Ghana involving 3183 women. The presence or absence of depressive symptoms within the past 30 days was estimated from the average score on three common symptoms of depression: sleep, anxiety, and sadness. The explanatory variables were age-group, socioeconomic level, marital status, ethnicity, religion, education, employment, and parity. Frequencies and means were used to summarize categorical and continuous variables, respectively. Logistic regression analyses were employed to determine the predictors of depressive symptoms.

**Results:**

The prevalence of depressive symptoms within the previous 30 days was 26.5% (95% CI: 25.0–28.1). Women 55 years and older were more likely than women between the ages of 18 and 24 to experience depressive symptoms (AOR 2.8, 95% CI: 2.0–4.0, *p* < 0.001), whilst women between the ages of 35 and 54 were 1.95 times more likely than women between the ages of 18 and 24 to experience depression (AOR 1.95, 95% CI: 1.40–2.70, *p* < 0.001). Self-employed women were less likely to report depressive symptoms compared to the unemployed (AOR 0.70, 95% CI: 0.56–0.87, *p* < 0.01). Akans were less likely to experience depressive symptoms compared to Ga women (AOR 0.75, 95% CI: 0.61–0.92, *p* < 0.01). Non-orthodox Christians were more likely to report depressive symptoms compared to Orthodox Christians (AOR 1.32, 95% CI: 1.09–1.60, *p* < 0.01).

**Conclusion:**

The prevalence of symptoms of depression among healthy urban Ghanaian women is high. Older women, those with low education, and unemployed women appear to be at higher risk for depression and therefore should be targeted for interventions. Groups at risk for depression—especially older adults or individuals under economic strain—should be targeted for mood assessment as part of routine medical care.

## Background

Depression is one of the most debilitating mental health disorders in the world. According to the World Health Organization (WHO), 350 million people worldwide suffer from depression, and it is a major contributor to the overall global burden of disease [1]. Unipolar depression has been identified as the second highest illness burden, accounting for as much as 8.6% of disability-adjusted life years lost, and is the single most important cause of disease-related disability among women in the reproductive age-group [2].

The literature is replete with data on the higher prevalence of depression among women [3]. Several factors explain this phenomenon, including sociodemographic and economic factors. Poverty, problems of conflict, and violence are higher among females and are associated with mental health problems and depression. Whilst some studies in Ghana have suggested a lack of association between marital status and depression [4, 5] others have reported that a significant proportion of the depressed are married women. Women in either abusive relationships or married to controlling husbands have higher odds of psychological distress [6]. There are also age correlates in reported depression. Among younger women, levels of depression are lower than among older women [7]. Although depression occurs at a much younger age among African women (between 20 and 40 years of age) compared to Europeans (between 35 and 55 years of age), depression is the most common mental health disorder among older men and women in Ghana aged 60 years and above [5]. These differences extend beyond menopause [8].

Education and employment are protective against depression and mental disorders in general. The risk of a common mental disorder is higher for the poor, the unemployed, individuals with low education, and the physically ill [3–5, 9, 10], although this finding has not always been consistent [11]. According to Canavan and colleagues [10], the lack of consistency among studies is largely due to dissimilarities in study designs within low and middle income countries (LMIC). This, in part, has accounted for our limited knowledge about the extent of the association between mental health and income. Few studies have been conducted on the prevalence of mental disorders in LMIC, and even fewer studies exist on specific mental disorders, such as depression. The majority of studies largely focus on identifying predictors or the treatment of depression [12, 13]. Most prevalence studies on mental health have used a small number of study subjects or are based at the facility level [14].

In most studies, researchers study clinical depression and are therefore likely to miss individuals who are burdened or who show minor signs of distress and depression that are not significant enough to be debilitating or to report to the hospital. Our primary objective was to measure the prevalence of self-reported depressive symptoms within the previous 30 days and to identify socioeconomic and sociodemographic factors associated with depressive symptoms among women in Accra in a large-scale study within an urban population. This study explores depressive symptoms among healthy women with diverse characteristics, allowing us to examine several background characteristics, including education and employment, as well as less examined characteristics, such as ethnicity and religion.

## Method

The data for this paper were obtained from the Women’s Health Study of Accra, Wave 1 (WHSA-1), a large-scale, cross-sectional study conducted in Accra, Ghana between 2003 and 2007 involving 3183 women aged 18 years and over. The study design is a population-based cross-sectional survey. Respondents were selected from the six sub-metropolitan areas that make up urban Accra. Accra is the capital of Ghana and is part of the Greater Accra Region, which includes the sub-metropolitan areas of Ablekuma, Ashiedu Keteke, Osu Klottey, Kpeshie, Ayawaso, and Okaikoi.

The sample consists of women 18 years and older who were randomly selected from enumeration areas categorized by socioeconomic status. Sampling was stratified by socioeconomic status and then by age. Older women above 55 years were over-sampled to increase their representation and to improve statistical power in the study. The selected women were administered a comprehensive household survey that included questions on general health (including sleep and feelings), risk factors for non-communicable diseases, physical activity, food security, reproductive health, sexual behaviour, health seeking behaviour, and family planning. A detailed description of the study variables and sampling procedures has been published elsewhere [15].

### Measurement

**Fig. 1:**
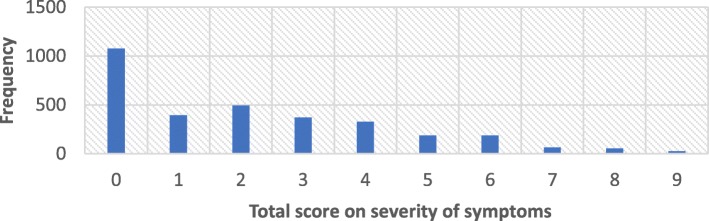
Distribution of depressive symptoms

The main outcome variable—the presence or absence of depressive symptoms—was derived from three primary questions that asked about common symptoms of depression: feelings of sadness or depression, sleep disturbances, and excessive worry. These were part of a set of items that measured mental health and well-being among the study participants. The items selected from the WHSA-1 have not been validated as a measure of depression for use in Ghana. Therefore, no clinical benchmark has been established on this scale to distinguish the severity of symptoms. While we are mindful of this limitation, it is important to note that the selected items are commonly associated with depression. Our interest was to obtain a measure that reflected presence or absence of depressive symptoms and therefore recalculated a composite score from the selected items. Each item was originally rated on a 4-point Likert scale: 0 (no symptoms), 1 (mild symptoms), 2 (moderate symptoms) and 3 (severe symptoms). The aggregated score of all three questions was calculated, and the mean was subsequently calculated to represent the score for depressive symptoms. Thus, an individual who reported severe symptoms on all three variables scored a total of 9 and a maximum mean of 3. The mean symptom score was re-coded into a binary outcome. Individuals with a mean symptom score of less than or equal to 1.0 were coded as not having depressive symptoms. Individuals with a mean symptom score of more than 1.0 were coded as having depressive symptoms. This binary outcome variable was used for chi square tests and logistic regression analyses. The distribution of scores is presented in Table 1 and in Fig. 1 below.

**Table 1:** Distribution of depressive symptoms scores

*Depressive Symptoms Total Scores	Frequency	Percent
0	1078	33.9
1	395	12.4
2	494	15.5
3	372	11.7
4	327	10.3
5	186	5.8
6	186	5.8
7	65	2.0
8	54	1.7
9	26	0.8
Total	3183	100

*Total scores were obtained from aggregate scores on three depressive symptoms. Sample questions include: Q1-How much of a problem did you have with sleeping, such as falling asleep, waking up frequently during the night, or waking up too early in the morning? (0 = None, 1 = Mild, 2 = Moderate, 3 = Severe or extreme). Q2-In the last 30 days, how much of a problem did you have with feeling sad, low or depressed? (0 = None, 1 = Mild, 2 = Moderate, 3 = Severe or extreme). Q3-In the last 30 days, how much of a problem did you have with worry or anxiety? (0 = None, 1 = Mild, 2 = Moderate, 3 = Severe or extreme)

### Analytic strategy

The data were checked for internal consistency and completeness using simple summary statistics of the selected variables and analysed in STATA 13.0 (Stata Corporation, College Station, Texas).

The explanatory variables were age-group, the enumerated socioeconomic level of the area of residence, marital status, ethnicity, religion, education, employment, and parity. Two continuous variables, age and parity, were categorized into four (4) and three (3) ordered groups, respectively. The following categorical variables were recoded into fewer categories to minimize the imbalance in counts in different categories. Ethnicity originally had 9 categories and was re-categorized into 5. Religion originally had 12 categories and was re-categorized into 4. Employment status originally had 9 categories and was re-categorized into 5.

Simple frequencies were used to describe categorical variables, whilst means and standard deviations were used to summarize continuous data. To predict the likelihood of or absence of depressive symptoms, a logistic regression analysis was conducted.

Some explanatory variables were found to be significant predictors of depressive symptoms in the crude analysis (*p* < 0.1). These were then used in the adjusted model to determine the effect of exposure variables on depressive symptoms after controlling for the effect of all other predictor variables at a 95% confidence interval and a significance level of 0.05.

## Results

### Descriptive characteristics of study participants

In total, 3183 women aged 18 and above were involved in this study, with a median age of 34.4 years. Over half of the study participants were less than 35 years old. Almost a third of the women were residents of low socioeconomic areas in the Greater Accra region. Over 40.0% had reached schooling of the Junior High School/Middle School level, the equivalent of approximately 10 years of education. Over one third of the respondents (38.1%) were married. These statistics are presented in detail in Table 2.

**Table 2:** Socio-demographic characteristics of respondents and associated chi square tests

	Depressive Symptoms				Total	%	Chi	*p*-value
	Absent		Present					
	N	%	N	%				
Level of education								
No education	469	20.2	248	29.6	717	22.7		
Primary	252	10.8	132	15.8	384	12.1		
Middle /JSS	1023	44.0	309	36.9	1332	42.1		
Secondary	401	17.3	107	12.8	508	16.1		
Tertiary	179	7.7	42	5.0	221	7.0		
Total	2324	100	838	100	3162	100	57.84	< 0.001
Socio-economic level of the enumeration area of residence								
Low Class	708	30.3	270	32.0	978	30.8		
Low Middle Class	530	22.7	214	25.4	744	23.4		
Upper Middle Class	548	23.5	195	23.1	743	23.4		
High Class	550	23.5	164	19.5	714	22.5		
Total	2336	100	843	100	3179	100	7.16	0.067
Marital status								
Never married	710	30.8	172	20.5	882	28.0		
Currently	918	39.8	280	33.4	1198	38.1		
Widowed/	588	25.5	350	41.8	938	29.8		
Ever married	91	3.9	36	4.3	127	4.0		
Total	2307	100	838	100	3145	100	84.4	< 0.001
Survey age-groups								
18–24	707	30.3	139	16.5	846	26.6		
25–34	626	26.8	191	22.7	817	25.7		
35–54	569	24.4	217	25.7	786	24.7		
55+	431	18.5	296	35.1	727	22.9		
Total	2333	100	843	100	3176	100	123.92	< 0.001
Religion								
Orthodox	894	38.3	297	35.2	1191	37.5		
Non-orthodox	953	40.9	354	41.9	1307	41.2		
Muslim	312	13.4	108	12.8	420	13.2		
Others	173	7.4	85	10.1	258	8.1		
Total	2332	100	844	100	3176	100	7.33	0.062
Ethnicity								
Ga	800	34.3	368	43.7	1168	36.8		
Akan	881	37.8	239	28.4	1120	35.3		
Ewe	300	12.9	117	13.9	417	13.1		
Northern	203	8.7	61	7.2	264	8.3		
Others	147	6.3	58	6.9	205	6.5		
Total	2331	100	843	100	3174	100	32.7135	< 0.001
Employment Status over the past 12 months								
Unemployed	434	18.6	259	30.8	693	21.8		
Formal employment	257	11	78	9.3	335	10.6		
Self employed	1219	52.3	409	48.7	1628	51.3		
Student/ apprentice	308	13.2	57	6.8	365	11.5		
Others (Housewife/retired/Volunteer)	115	4.9	37	4.4	152	4.8		
Total	2333	100	840	100	3173	100	68.0365	< 0.001
Parity								
	194	8.3	85	10.1	279	8.8		
1 to 3 children (1–3)	863	36.9	293	34.7	1156	36.3		
4 or more children	1282	54.8	466	55.2	1748	54.9		
Total	2339	100	844	100	3183	100	3.0645	0.216

Of the total sample, 844 women reported depressive symptoms within the previous 30 days, with a prevalence of 26.5% (95% CI: 25.0–28.1). Table 1 shows the distribution of scores of the aggregated depressive symptoms. Chi square tests for trends between categorical variables (Table 2) revealed that the level of education, marital status, age-group, ethnicity and employment status within the previous 12 months were strongly associated with depressive symptoms (*p* <  0.001). The number of children a woman had, her religious affiliation, and the socioeconomic status of her area of residence had no association with the depressive symptoms by chi square test (*p* > 0.05).

The results of a univariable logistic regression analysis indicated that age-group, the enumerated socioeconomic level of the area of residence, the level of education, marital status, employment, and religion were significantly associated with depressive symptoms. After adjusting for the effect of all other variables, the following predictors of depressive symptoms were identified: age-group, educational level, occupation, religion, and ethnicity (Table 3). In the adjusted model, other variables, such as marital status, the socioeconomic level of the area of residence, and parity, were not significant predictors of depressive symptoms within the previous 30 days.

**Table 3:** Logistic regression analysis of the association between depressive symptoms and sociodemographic characteristics of participants (*N* = 3183)

Characteristics	Crude OR (95% CI)	*p*-value	Adjusted OR (95% CI)	*p*-value
Age-group				
18–24 (reference)	1		1	
25–34	1.55(1.2–1.98)	< 0.001*	1.70 (1.26–2.28)	< 0.001
35–54	1.94 (1.53–2.47)	< 0.001*	1.95 (1.40–2.70)	< 0.001
55+	3.49 (2.76–4.41)	< 0.001*	2.82 (1.96–4.04)	< 0.001
Socio economic level of the enumeration area of residence				
Low (reference)	1		1	
Lower Middle Class	1.06 (0.86–1.31)	0.597	1.1 (0.87–1.37)	0.435
Upper Middle Class	0.93 (0.75–1.16)	0.528	0.96 (0.76–1.21)	0.751
High Class	0.78 (0.63–0.98)	0.031*	0.85 (0.66–1.08)	0.193
Level of Education				
No Education (reference)	1		1	
Primary	0.99 (0.76–1.28)	0.943	1.37 (1.03–1.83)	0.029*
Middle/JHS	0.57 (0.47–0.70)	< 0.001*	0.81 (0.64–1.03)	0.091
Secondary/SSS	0.50 (0.38–0.66)	< 0.001*	0.85 (0.62–1.17)	0.310
Higher	0.44 (0.31–0.62)	< 0.001*	0.69 (0.46–1.05)	0.085
Marital status				
Never married (reference)	1		1	
Currently married	1.26 (1.02–1.56)	0.035*	0.76 (0.57–1.03)	0.074
Widowed/Divorced/ Separated	2.46 (1.99–3.04)	< 0.001*	1.08 (0.78–1.50)	0.654
Ever Married, Current Status Unknown	1.63 (1.07–2.49)	0.022*	0.79 (0.49–1.29)	0.348
Ethnicity				
Ga (reference)	1		1	
Akan	0.59 (0.49–0.71)	< 0.001*	0.75 (0.61–0.92)	0.006*
Ewe	0.85 (0.66–1.09)	0.190	1.0 (0.78–1.3)	0.938
Northern Descent	0.65 (0.48–0.89)	0.007	0.87 (0.57–1.3)	0.503
Others	0.87 (0.62–1.19)	0.359	1.13 (0.73–1.74)	0.581
Religion				
Orthodox Christian (reference)	1		1	
Non-orthodox Christian	1.11 (0.93–1.34)	0.222	1.32 (1.09–1.60)	0.005
Muslim	1.04 (0.80–1.34)	0.752	0.97 (0.66–1.42)	0.886
Others	1.47 (1.11–1.99)	0.008*	1.30 (0.93–1.73)	0.137
Employment				
Unemployed (reference)	1		1	
Formal employment	0.51 (0.38–0.68)	< 0.001	0.79 (0.57–1.10)	0.163
Self-employed	0.56 (0.47–0.68)	< 0.001	0.70 (0.56–0.87)	0.001*
Student /Apprenticeship	0.31 (0.22–0.43)	< 0.001	0.68 (0.46–0.99)	0.044*
Others (Housewife/retired/Volunteer, etc.)	0.54 (0.36–0.81)	< 0.001	0.61 (0.40–0.92)	0.019*
Parity				
No children (reference)	1		1	
1 to 3 children (1–3)	0.77 (0.58–1.03)	0.082	1.04 (0.76–1.44)	0.796
Four or more children (4+)	0.83 (0.63–1.09)	0.185	1.02 (0.76–1.37)	0.897

*OR* Odds ratio, *CI* Confidence interval

*significant results

Age group remained a very important predictor of depressive symptoms. Older women had increased odds of experiencing depressive symptoms compared to younger women between the ages of 18 and 24. After adjustment, women 55 years and above were 2.8 times more likely than women within the 18 to 24 age group category to experience depressive symptoms (AOR 2.8, 95% CI: 2.0–4.0, *p* < 0.001), whilst women between the ages of 35 and 54 were 1.95 times more likely than women between the ages of 18 and 24 years to experience depressive symptoms (AOR 1.95, 95% CI: 1.40–2.70, *p* <  0.001). On the other hand, the odds of experiencing depressive symptoms among women between 25 and 34 years of age were 1.7 times greater than among women between 18 and 24 years of age (AOR 1.7, 95% CI: 1.3–2.30, *p* < 0.001).

Employment remained an important predictor of depressive symptoms in the adjusted model. Self-employed women were 30% less likely to report depressive symptoms (AOR 0.70, 95% CI: 56.1–86.7, *p* < 0.01) compared to unemployed women. The odds of students/apprentices reporting depressive symptoms were 0.68 times as great as unemployed women (AOR 0.68, 95% CI: 0.46–0.99], *p* < 0.05). Although women in formal employment were 21% less likely to experience depressive symptoms compared to unemployed women, this could have been attributed to chance (*p* > 0.05).

In Ghana, religion plays an important role in depression. Non-orthodox Christians were significantly more likely to report depressive symptoms within the previous 30 days compared to orthodox Christians (AOR 1.32, 95% CI: 1.09–1.60, *p* < 0.01). Women who shared other minority religious faiths (traditionalists, spiritualists, and atheists) were 1.47 times more likely to report depressive symptoms compared to orthodox Christians (*p* < 0.01).

No statistically significant associations were observed across the other religious groupings. The odds of experiencing depressive symptoms among Akan women were 0.75 times as great as among Ga women (AOR 0.75, 95% CI: 0.61–0.92, *p* < 0.01).

The adjusted model revealed no association between marital status and depression, even though the crude analysis showed a protective effect from the reduced odds of depression enjoyed by women who never married compared to women in other relationships.

Women with primary education were 1.4 times more likely to report depressive symptoms compared to women with no formal education. Although women who had schooled beyond the primary level were less likely to report depressive symptoms, the strength of the evidence was rather weak in the adjusted analysis (*p* > 0.05).

## Discussion

A high prevalence of depressive symptoms was observed among the healthy participants, and depressive symptoms increased with age. Self-employed women or women engaged as students or apprentices had reduced odds of reporting depressive symptoms compared to unemployed women. Being an orthodox Christian appeared to offer protection against depressive symptoms. Despite the strong association between age, employment, and depressive symptoms, we caution against the assumption of causality. Proving causality in cross-sectional studies is statistically and empirically difficult.

We found a relatively higher prevalence of depressive symptoms in our study than has previously been reported [6, 11, 16–19]. The only exception we are aware of is the 41.1% figure reported for Vietnam [18]. One reason for this may have been the relatively few items used in our assessment of depressive symptoms. We distinguished depressive symptoms from clinical depression that would require a stricter criterion, as specified in the DSM 5 or ICD-10. Even so, the assessment of depressed mood, sleep disturbance, and worry or anxiety are prominent symptoms of depression and are usually adversely affected during depression. Therefore, the proportion of people who might show clinically relevant signs of depression and yet would not consider the symptoms serious enough to seek treatment is interesting. These early signs of depression may be due to high levels of stress associated with the hassles of urban living. Much evidence exists showing strong links between poor mental health and stress or urban living [20, 21]. Other studies have shown an increase in nuclear families in urban areas, which in turn is associated with increased violence among women and the mental health of poor women [19]. In other words, the social safety net provided by the extended family in less urban areas is either diminished or removed, exposing vulnerable individuals to high stress levels, possibly due to challenging economic circumstances.

Our results support earlier studies on the association between age and depression. For example, Deyessa and colleagues [11] showed that the odds of experiencing depression increase with age. Compared to younger women, older individuals have more anxieties stemming from multiple sources: marriage, children, stable employment, and income. Older unmarried women have more worries about marriage and childbirth, motivated by societal pressures on marriage, [22] particularly in Africa where marriage and motherhood are expected of every woman.

In the crude analysis—compared to women who never married—unmarried women (divorced, separated, or widowed) had significantly higher odds of reporting depressive symptoms. This finding supports earlier reports by others [11]. Arguably, these life changes, in the absence of adequate social support, will present substantial stress that can induce depression.

Contrary to what has been reported previously, we did not find an association between socioeconomic status and depression, despite adequate global reporting of this effect [4, 5, 23]. For example, a large scale longitudinal study in Belgium reported that increases in financial strain, poverty, and the end of cohabitation were associated with increased depressive symptoms [23]. We found some of these in our study, but our measure of socioeconomic status was based on the respective locality or census enumeration areas within which we selected our respondents. A lack of variability in the enumeration areas likely limited the different characteristics that might contribute to socioeconomic status. In Nigeria, a higher prevalence has been reported among rural dwelling people than among urban inhabitants [24]. While we did not compare between rural and urban populations, rural societies are associated with lower education and income, both of which are indicators of lower socioeconomic status. The measure of socioeconomic status is not consistent in many instances and that should be noted in future research.

We found a limited effect of ethnicity and religion on depressive symptoms; that is, the effect was reported only for specific groups, such as Akans and non–orthodox Christians. Compared to indigenous Ga women, the reduced odds of depression observed among women of Akan extraction in both the crude and the adjusted model are interesting and present an interesting subgroup worth exploring in future research.

The major finding of this study is the realization that more self-reported depressive symptoms, or mild forms of depression, might exist in supposedly healthy populations than has previously been reported. This has important implications for public health policy. In routine examinations, we should begin including an assessment of mood disorders among selected groups, especially among older adults or individuals under economic strain. The usefulness of early detection of mental health problems cannot be overemphasized. With this in mind, we can begin to institute referrals and interventions following early detection and diagnosis of depression. Counselling and psychological services are limited and expensive globally. In many countries, particularly LMICs, they are not covered by health insurance schemes, thereby making them inaccessible. When counselling and psychological services are placed in the public health domain, provision of care is easily accessible.

### Limitations

This study is not without limitations.

First, it is a cross-sectional study and therefore, as we have stated earlier, is limited in making any causal associations between the predictors and prevalence of depressive symptoms. One issue that should be clarified is the definition of depression as distinctly different from clinical depression. Measurement of clinical depression is much more stringent and usually follows criteria determined by DSM 5 or ICD 10. Our assessment of depression involved a self-reported assessment of the degree of disturbance in three symptoms usually associated with depression within 1 month. Most depression inventories are more detailed. We also acknowledge that self-reported measures are not as robust as objective assessments of behaviour. Self-reported data also have issues with recall bias. Diseases associated with mental health are stigmatized in Ghana; therefore, subjects may be tempted to hide their depression status.

Second, socioeconomic status was measured based on residence, an assumption supported by data from the Ghana Statistical Service enumeration data. We have reported previously that this is limited. Despite this, there is very little unanimity in the measurement of socioeconomic status across studies.

## Conclusion

The prevalence of depressive symptoms among urban Ghanaian women is relatively high. This means that the reported risk burden of women’s mental health is substantially underestimated. This finding is also true for individuals who are older, unemployed, or for those with low education. We found a higher prevalence among older individuals than among younger individuals. Among specific groups, the unemployed and those with lower education also showed susceptibility to depression. We advocate further research on high risk groups, such as the elderly, those under the strain of poverty, the unemployed, and the lowly educated. We expect that increased knowledge on this would provide new directions in public health policy that could facilitate early detection and referral of people found to be at risk.

## Abbreviations

DSM 5: Diagnostic Statistical Manual 5th Edition
ICD 10: International Classification of Disorders 10th Edition
LMIC: Low and Middle-Income Countries
WHO: World Health Organization
WHSA-1: Women’s Health Study of Accra, Wave 1
